# Phylogeny of Anoxygenic Photosynthesis Based on Sequences of Photosynthetic Reaction Center Proteins and a Key Enzyme in Bacteriochlorophyll Biosynthesis, the Chlorophyllide Reductase

**DOI:** 10.3390/microorganisms7110576

**Published:** 2019-11-19

**Authors:** Johannes F. Imhoff, Tanja Rahn, Sven Künzel, Sven C. Neulinger

**Affiliations:** 1 GEOMAR Helmholtz Centre for Ocean Research, 24105 Kiel, Germany; trahn@geomar.de; 2 Max Planck Institute for Evolutionary Biologie, 24306 Plön, Germany; kuenzel@evolbio.mpg.de; 3 omics2view.consulting GbR, 24118 Kiel, Germany; s.neulinger@omics2view.consulting

**Keywords:** phylogeny, photosynthetic reaction center proteins, bacteriochlorophyll biosynthesis, phototrophic purple bacteria, evolution of anoxygenic photosynthesis

## Abstract

Photosynthesis is a key process for the establishment and maintenance of life on earth, and it is manifested in several major lineages of the prokaryote tree of life. The evolution of photosynthesis in anoxygenic photosynthetic bacteria is of major interest as these have the most ancient roots of photosynthetic systems. The phylogenetic relations between anoxygenic phototrophic bacteria were compared on the basis of sequences of key proteins of the type-II photosynthetic reaction center, including PufLM and PufH (PuhA), and a key enzyme of bacteriochlorophyll biosynthesis, the light-independent chlorophyllide reductase BchXYZ. The latter was common to all anoxygenic phototrophic bacteria, including those with a type-I and those with a type-II photosynthetic reaction center. The phylogenetic considerations included cultured phototrophic bacteria from several phyla, including *Proteobacteria* (138 species), *Chloroflexi* (five species), *Chlorobi* (six species), as well as *Heliobacterium modesticaldum (Firmicutes)*, *Chloracidobacterium acidophilum (Acidobacteria),* and *Gemmatimonas phototrophica (Gemmatimonadetes)*. Whenever available, type strains were studied. Phylogenetic relationships based on a photosynthesis tree (PS tree, including sequences of PufHLM-BchXYZ) were compared with those of 16S rRNA gene sequences (RNS tree). Despite some significant differences, large parts were congruent between the 16S rRNA phylogeny and photosynthesis proteins. The phylogenetic relations demonstrated that bacteriochlorophyll biosynthesis had evolved in ancestors of phototrophic green bacteria much earlier as compared to phototrophic purple bacteria and that multiple events independently formed different lineages of aerobic phototrophic purple bacteria, many of which have very ancient roots. The *Rhodobacterales* clearly represented the youngest group, which was separated from other *Proteobacteria* by a large evolutionary gap.

## 1. Introduction

Anoxygenic photosynthesis is widely distributed among eubacteria and involves a number of genes for the photosynthetic reaction center and for the biosynthesis of photosynthetic pigments, bacteriochlorophylls, and carotenoids, which are essential elements to enable photosynthesis. While the biosynthesis of bacteriochlorophylls is common to all of them, the different structure of the photosynthetic reaction center clearly separates two groups of anoxygenic phototrophic bacteria, those having a type-I and those having a type-II photosystem [1,2,3].

Those bacteria employing a photosystem type-II photosynthetic apparatus include the phototrophic purple bacteria (*Proteobacteria*), as well as *Gemmatimonas* and *Chloroflexus,* with their photosynthetic relatives [1,3,4,5]. Essential components of the type-II photosynthetic apparatus are represented by two membrane-spanning photosynthetic reaction center proteins that are common to all of these bacteria. These PufLM proteins are binding bacteriochlorophyll molecules and are crucial components of the type-II photosynthetic apparatus. Together with an additional protein (PufH = PuhA), they form the core structure of the type-II photosynthetic reaction center in all phototrophic purple bacteria (*Proteobacteria* and *Gemmatimonas*). The PufH protein is absent from the *Chloroflexi* that have chlorosomes attached to the reaction center. In addition, a cytochrome c (PufC) is associated with the reaction center proteins in the majority of phototrophic purple bacteria but is lacking in a number of species [6]. While *pufLMC* genes form a stable genomic cluster (sometimes lacking the *pufC* gene), *pufH* (*puhA*) is located at a different place within the genome, associated with genes of bacteriochlorophyll biosynthesis [6]. It has been demonstrated that sequences of PufLM are excellent tools to study the phylogeny of anoxygenic phototrophic purple bacteria, as well as their diversity and environmental distribution [2,7,8,9]. In a comprehensive study based on the phylogeny of PufLM, it was shown that distinct lineages of *Proteobacteria* contained phototrophic representatives in 10 orders, including anaerobic as well as aerobic anoxygenic phototrophic purple bacteria [3].

Bacteriochlorophyll biosynthesis is common to all phototrophic bacteria, including those with a type-I and those with a type-II photosynthetic reaction center. A key enzyme in this pathway is the light-independent chlorophyllide reductase BchXYZ. Consequently, this protein enables a broad view on the phylogeny of anoxygenic photosynthetic bacteria with a capacity to synthesize bacteriochlorophyll [10].

**Figure 1:**
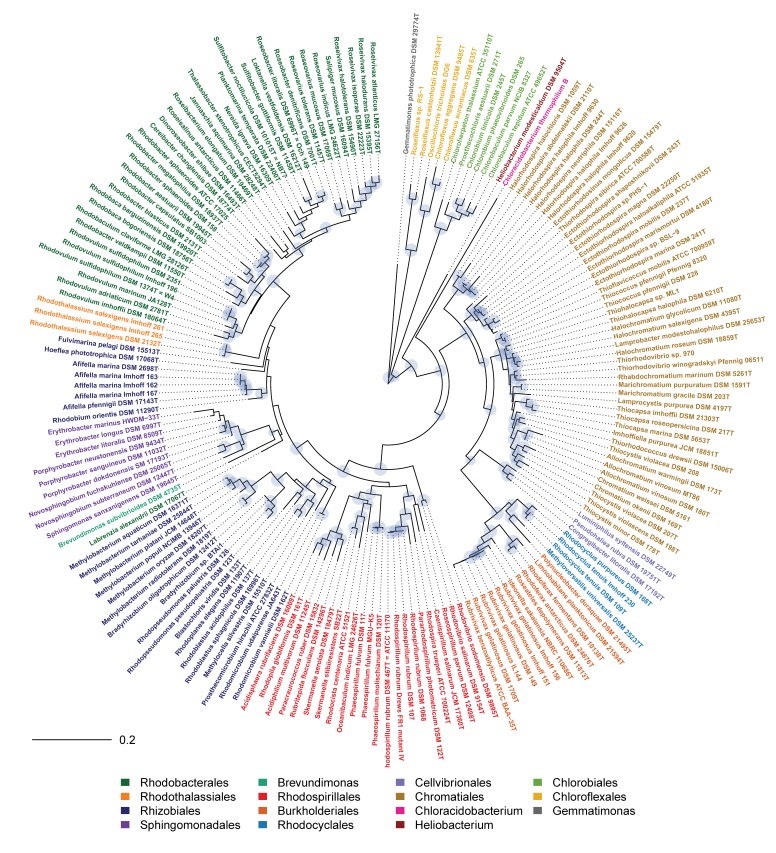
Phylogenetic tree (RNA tree) of phototrophic bacteria according to 16S rRNA gene sequences.

**Figure 2:**
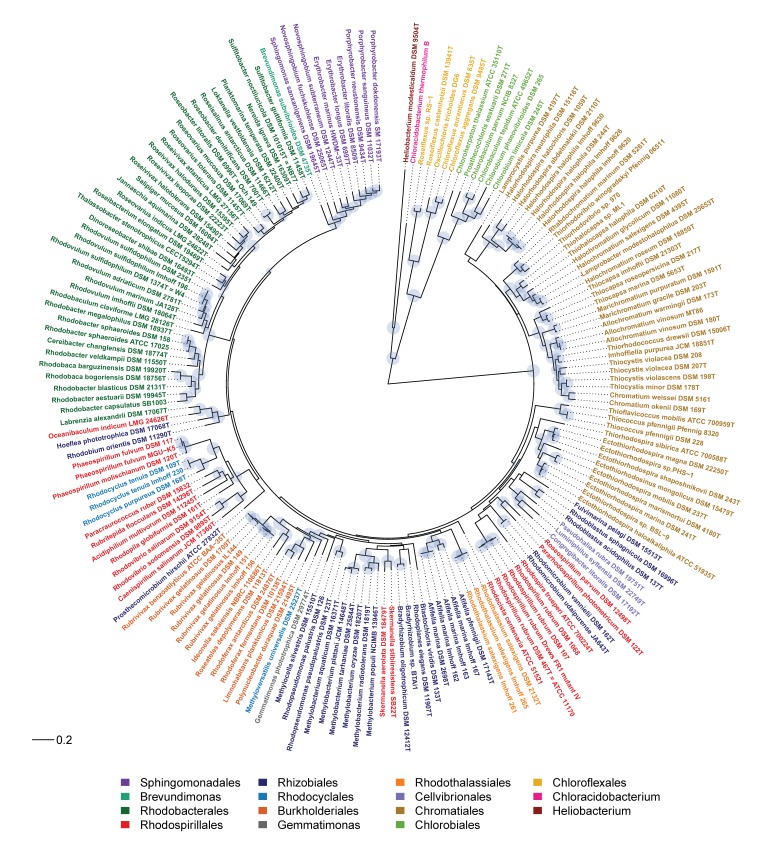
Phylogenetic tree of phototrophic bacteria, according to BchXYZ sequences.

**Figure 3:**
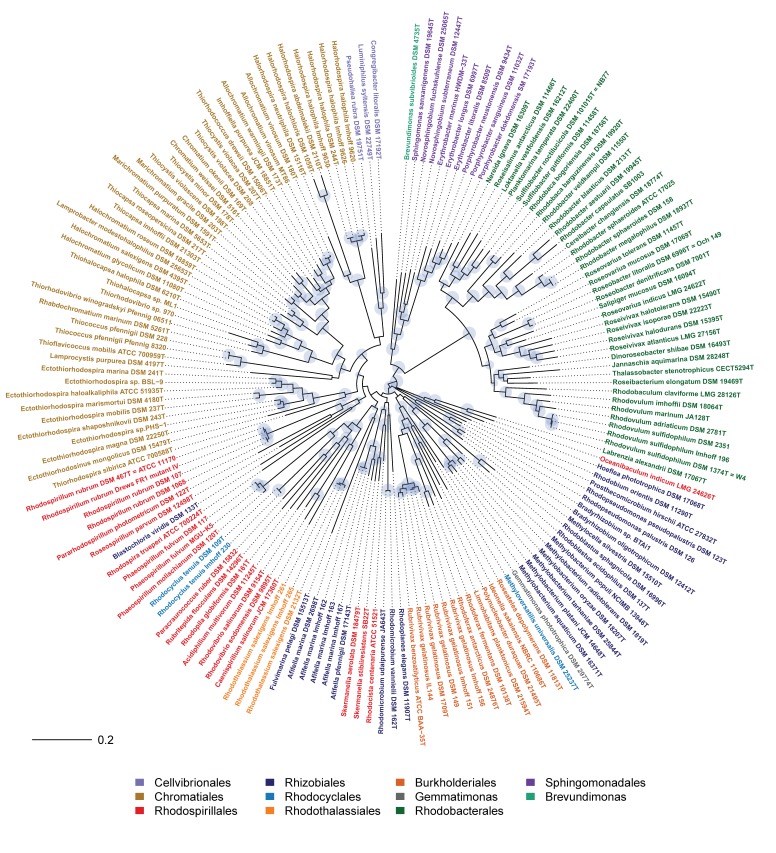
Phylogenetic tree (PS tree) of phototrophic bacteria, according to BchXYZ-PufHLM sequences.

**Figure 4:**
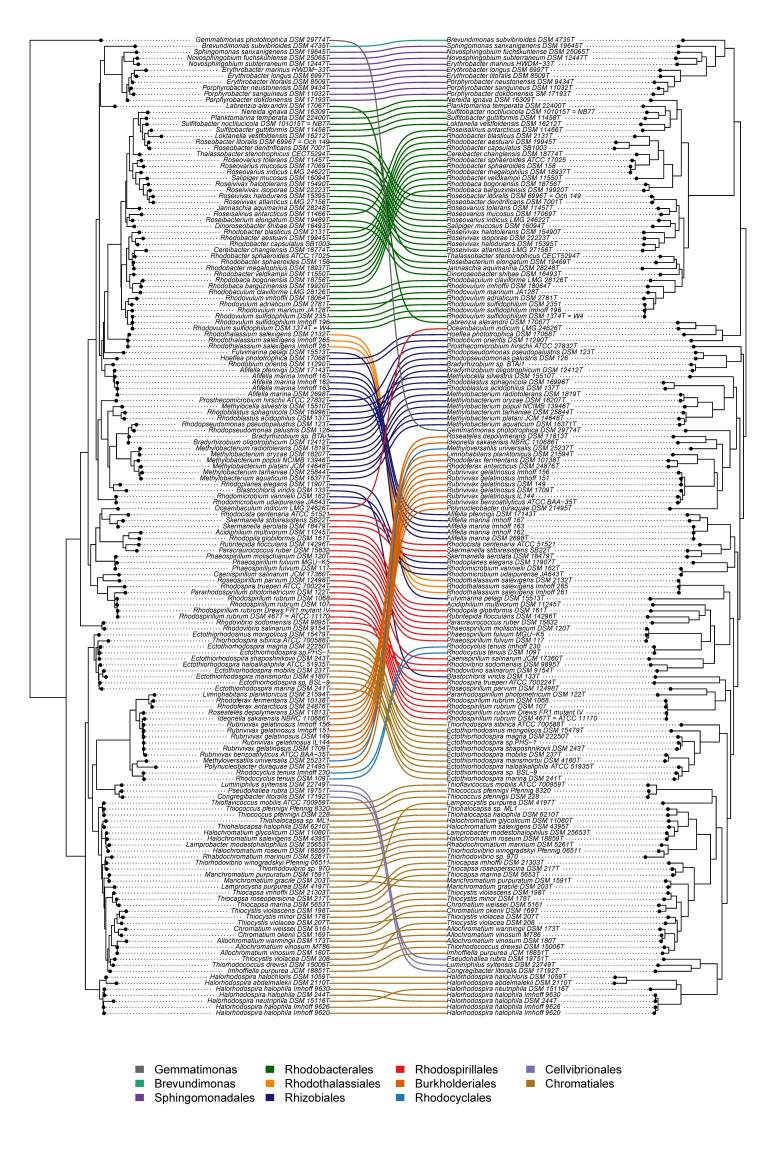
Linear comparison tree of 16S rRNA gene sequences (right) and BchXYZ-PufHLM sequences (left) of phototrophic bacteria.

In the present work, the phylogeny of anoxygenic phototrophic bacteria was analyzed on the basis of sequences of key proteins of the type-II photosynthetic reaction center PufHLM and of chlorophyllide reductase BchXYZ and was compared with the phylogeny of the 16S rRNA gene (Figure 1). The phylogenetic tree of BchXYZ (Figure 2) gave an overview of all considered strains, while that of combined sequences of PufHLM-BchXYZ (Figure 3) covered all considered phototrophic purple bacteria. In addition, phylogenies of combined PufHLM-BchXYZ sequences and 16S rRNA gene sequences were compared (Figure 4).

## 2. Material and Methods

### 2.1. Cultivation, Sequencing, and Assembly of DNA Sequences

Cells were grown in the appropriate media, as described for the purple sulfur bacteria [11,12] and several groups of phototrophic purple bacteria [13]. Extraction and sequencing of DNA and the assembly of sequences were described earlier [14].

### 2.2. Sequences

Sequences of PufL, PufM, PufH, PufC, BchXYZ were retrieved from the annotated genomes. Genome sequences were annotated using the rapid annotations using subsystems technology (RAST) [15,16]. All sequences were deposited in the EMBL database. Accession numbers, together with species and strain designations, as well as the corresponding higher taxonomic ranks, are included in Supplementary Table S1.

### 2.3. Phylogenetic Analyses

Multiple sequence alignments (MSAs) were produced with MAFFT v7.313 [17,18] from all sequences and were visually inspected for consistency. MAFFT was run with parameters ‘- globalpair - maxiterate 1000′. Alignment positions with >25% gaps were trimmed from MSAs. Maximum likelihood (ML) phylogenetic trees were calculated from MSAs with IQ-TREE v1.6.1 [19] using the best substitution models inferred from MSAs. For trees calculated from combined alignments (‘bchXYZ’ and ‘bchXYZpufHLM’), substitution models were used as so-called partition models [20]. Ultrafast bootstrap approximation (UFBoot) [21] was used to provide branch support values with 1000 replicates based on the same substitution models as the original ML tree. Branch support values were assigned onto the original ML tree as the number of times each branch in the original tree occurred in the set of bootstrap replicates (IQ-TREE option ‘-sup’).

Phylogenetic trees were midpoint-rooted and formatted using functionality from R packages ape v5.0.1 [22], phangorn v2.3.2 [23], and phytools v0.6.45 [24]. Bootstrap values within a range of 80–100% were visualized as filled circles. The circle area is a linear function of the respective bootstrap value. The scale bar beneath a tree indicates the number of substitutions per alignment site. A co-phylogenetic plot was produced to facilitate the comparison of selected phylogenies. Nodes of compared trees were rotated to optimize tip matching.

## 3. Results and Discussion

### 3.1. Strain and Sequence Selection

Representatives of phototrophic *Proteobacteria* (10 orders, 21 families, 86 genera, 138 species, 159 strains + five unclassified strains) together with five representatives of *Chloroflexi* (one order, three families, three genera, five species) and six selected *Chlorobi* (one order, one family, four genera, six species), as well as *Gemmatimonas phototrophica*, *Chloracidobacterium acidophilum,* and *Heliobacterium modesticaldum* were included in the phylogenetic analyses of this study.

Depending on the availability of gene and genomic information, primarily sequence information from the type and reference strains was considered. In order to avoid any incongruity due to strain-dependent sequence variation, sequences from identical strains were used for all phylogenetic trees. All species and strain numbers are presented in Supplementary Table S1.

### 3.2. Phylogeny According to 16S rRNA Gene Sequences

As the 16S rRNA gene is established as a phylogenetic reference since the pioneering work of Carl Woese [25], we included the phylogenetic tree of this gene showing the relationship of all strains selected for the present study (RNA tree, Figure 1) and later compared this phylogenetic relationship with that of key proteins of photosynthesis (Figure 4). Clearly separated and distinct major groups with the deepest branching points in the tree were represented by *Chlorobi*, *Chloroflexi*, as well as *Heliobacterium modesticaldum* (representative of *Firmicutes* phylum), *Chloracidobacterium thermophilum* (representative of *Acidobacteria* phylum), and *Gemmatimonas phototrophica* (representative of *Gemmatimonadetes* phylum) (Figure 1). Quite remarkable was the isolated position of *Gemmatimonas*, which encodes a typical proteobacterial photosynthetic apparatus [26,27].

The *Proteobacteria* formed two distinct major branches with all *Alphaproteobacteria* in one branch and the *Gammaproteobacteria* and *Betaproteobacteria* in another branch. In the *Gammaproteobacteria* branch, distinct lineages were represented by *Chromatiaceae*, the *Ectothiorhodospira* group, the *Halorhodospira* group, the *Cellvibrionales* (aerobic anoxygenic phototrophic *Gammaproteobacteria*), and the *Betaproteobacteria*.

A much more complex situation existed within the *Alphaproteobacteria,* with a number of small groups with larger phylogenetic distance. The *Rhodobacterales* and also core groups of *Rhodospirillales*, *Rhizobiales,* and *Sphingomonadales* formed well-supported branches, which were, however, poorly resolved in their relationship to each other. Supported branches were formed by the members of the following genera:

(i) Rhodospirillum, Roseospirillum/Caenispirillum, Rhodospira, Pararhodospirillum,
(ii) Phaeospirillum, Oceanibaculum, Rhodocista, Skermanella,
(iii) Rhodopila, Rubritepida, Paracraurococcus, Acidiphilum, Acidisphaera,
(iv) Erythrobacter, Porphyrobacter, Novosphingobium, Sphingomonas,
(v) Rhodopseudomonas/Bradyrhizobium, Methylobacterium.

Most remarkable were the isolated positions of representatives of *Fulvimarina*, *Hoeflea*, *Labrenzia*, *Rhodothalassium,* and *Afifella-Rhodobium.* Though distant to other phototrophic bacteria, *Brevundimonas* clearly was linked to the *Sphingomonadales* branch. In addition, *Rhodovibrio* species appeared as clear outsiders and formed the most deeply branching lineage within the *Alphaproteobacteria*. In addition, several small groups were formed by single species or a few species only. These included species of *Blastochloris*, *Rhodoplanes*, *Rhodoblastus*, *Methylocella*, *Prosthecomicrobium,* and *Rhodomicrobium* (Figure 1).

It should be emphasized that *Roseospirillum parvum* was associated with the *Rhodospirillaceae* and, in particular, with the *Rhodospirillum/Pararhodospirillum* group as also *Caenispirillum* and *Rhodospira trueperi* do (Figure 1), supporting the current taxonomic classification [28].

### 3.3. Phylogeny of Photosynthesis

In order to evaluate the phylogeny of the photosynthetic apparatus, sequences of essential proteins for photosynthesis were analyzed. These included the bacteriochlorophyllide reductase BchXYZ and the photosynthetic reaction center proteins PufHLM and PufC. While the phylogenetic tree of BchXYZ (Figure 2) gave an overview of all considered strains and included all of the phototrophic green bacteria, the tree with combined sequences of PufHLM-BchXYZ (Figure 3) covered all phototrophic purple bacteria. PufC sequences were not considered in these trees because this component was absent from a number of representative species. All sequences and their accession numbers are presented in Supplementary Table S1.

#### 3.3.1. Phylogeny according to BchXYZ Sequences

The phylogeny of BchXYZ allows the widest view on the phylogeny of photosynthesis in phototrophic bacteria, including PS-I and PS-II bacteria. The chlorophyllide reductase BchXYZ catalyzes the first step in bacteriochlorophyll biosynthesis that differentiates this pathway from the biosynthesis of chlorophyll. It is present in all phototrophic bacteria producing different forms of bacteriochlorophyll [10].

The deepest and likewise most ancient roots according to BchXYZ sequences (Figure 2) were found in the phototrophic green bacteria that employ a type-I photosystem, the *Chlorobi*, *Heliobacterium modesticaldum* and relatives, and *Chloracidobacterium thermophilum,* as well as in *Chloroflexi* that employ a type-II photosystem (like all *Proteobacteria*). The large sequence differences to the phototrophic purple bacteria pointed out that bacteriochlorophyll biosynthesis had evolved in ancestors of green bacteria much earlier as compared to phototrophic purple bacteria. This relationship quite well correlated to the phylogeny of the 16S rRNA gene (RNA tree) (Figure 1), with the exception of *Gemmatimonas phototrophica*, which, according to BchXYZ, was distantly associated with the *Betaproteobacteria*, specifically the *Burkholderiales* with *Rubrivivax* and *Rhodoferax* as representative genera. The phylogeny of photosynthesis in *Proteobacteria* was discussed on the basis of more comprehensive information of the BchXYZ-PufHLM sequences below (Figure 3).

#### 3.3.2. Phylogeny of BchXYZ-PufHLM and Comparison with 16S rRNA Phylogeny

The combined sequence information of the key proteins of the photosynthetic reaction center in photosystem-II bacteria (PufHLM) and of the bacteriochlorophyll biosynthesis with the subunits of the chlorophyllide reductase (BchXYZ) gave a solid basis (alignment length, 2458 aa) to trace back the phylogeny of photosynthesis within the phototrophic purple bacteria (Figure 3). The consideration of PufHLM excluded the *Chloroflexi* (they lack PufH) in this consideration and restricted the view to *Proteobacteria* and *Gemmatimonas*. A direct comparison of the comprehensive phylogeny of anoxygenic photosynthesis, including sequences of BchXYZ-PufHLM (PS tree), with the phylogenetic relations according to 16S rRNA gene sequences (RNA tree) enlightened the evolution of photosynthesis as compared to that of the protein-producing machinery (Figure 4).

##### *Gammaproteobacteria (Chromatiales and Cellvibrionales)* 

The phototrophic *Gammaproteobacteria* represented a well-established major phylogenetic branch with four major sub-branches, which were well supported within both PS tree and RNA tree. The sub-branches included

(i) the Halorhodospira species,
(ii) the *Ectothiorhodospiraceae,* including *Ectothiorhodospira* and *Ectothiorhodosinus* species, with *Thiorhodospira* being associated more distantly, but excluding the *Halorhodospira* species,
(iii) the *Chromatiaceae* with subgroups of a) the *Thiococcus* group of bacteriochlorophyll-b containing *Chromatiaceae,* including species of *Thiococcus* and *Thioflavicoccus*; b) the *Halochromatium* group with halophilic species of the genera *Halochromatium*, *Lamprobacter*, *Rhabdochromatium*, *Thiorhodovibrio*, and *Thiohalocapsa*; c) the *Chromatium group* with species of *Chromatium*, *Thiocapsa*, *Marichromatium*, *Allochromatium*, *Thiorhodococcus*, *Imhoffiella*, and *Thiocystis*; d) *Lamprocystis purpurea* as an outsider among the *Chromatiaceae* with distant relationship to others and no statistical support of its position. Most significantly, *Lamprocystis purpurea* formed a deeply branching line in the *Chromatium*-group according to both 16S rRNA phylogeny and PS phylogeny. Therefore, it is likely to be an ancient representative of the *Chromatiaceae,*
(iv) the Cellvibrionales (Haliaceae) with Congregibacter litoralis, Luminiphilus syltensis, and Pseudohaliea rubra (most likely including Chromatocurvus halotolerans [3]), which were linked with low confidence to the Halorhodospira group. The Cellvibrionales formed a group distant to other Gammaproteobacteria according to both trees. In the RNA tree, they were linked to the Betaproteobacteria (in this tree within the frame of the Gammaproteobacteria), and in the PS tree, associated with the Halorhodospira group. Apparently, they represent an ancient phylogenetic lineage of the Gammaproteobacteria without clearly resolved roots.

It was remarkable that the species with bacteriochlorophyll-b, according to the PS tree, formed different deeply rooted lineages associated with the corresponding bacteriochlorophyll-a containing relatives, *Hlr. abdelmalekii* and *Hlr. halochloris* were associated with the *Halorhodospira* branch, *Thiorhodococcus* and *Thioflavicoccus* species with the *Chromatiaceae*, and *Rhodospira trueperi* and *Blastochloris viridis* with the *Rhodospirillaceae,* specifically with the *Rhodospirillum* group though with a low significance (Figure 3). The incorporation of all bacteriochlorophyll-b-containing bacteria within one common cluster is restricted to the phylogeny of the reaction center proteins PufLM [3]. This has been previously explained by the congruent evolution of the reaction center proteins with respect to the specific binding requirements of the bacteriochlorophyll-b molecule [3] and implicates the independent evolution of the photosystems with bacteriochlorophyll-b in the different phylogenetic lineages.

##### *Betaproteobacteria* (*Burkholderiales* and *Rhodocyclales*)

One of the most obvious differences between PS and RNA trees was in the position of the *Betaproteobacteria*. In the RNA tree, *Rhodocyclales* and the *Burkholderiales* formed two related lineages of a major branch within the frame of the *Gammaproteobacteria* (Figure 1). In the PS tree, both groups formed clearly separated clusters, which were associated with different branches of the *Alphaproteobacteria* (Figure 3). The *Burkholderiales* formed a deep and not safely rooted branch, including separate lineages of *Rubrivivax, Ideonella/Roseateles, Rhodoferax/Limnohabitans*, *Polynucleobacter,* and *Methyloversatilis.* The deep roots identify the photosynthesis of these bacteria as very ancient and, despite the poorly supported branches, could indicate a possible acquisition of photosynthesis by gene transfer from an early phototrophic alphaproteobacterium, as supposed earlier [6,29] (Igarashi et al., 2001; Nagashima and Nagashima, 2013). The whole group was also visible in the RNA tree but associated with the *Gammaproteobacteria*. In the PS tree, *Rhodocyclus* was linked to *Phaeospirillum* and the *Rhodospirillales* (Figure 3 and Figure 4), contrasting its link to the *Burkholderiales* in the RNA tree (Figure 1). This change might be indicative of a single event of a transfer of the photosynthesis genes from an ancient alphaproteobacterium within the *Rhodospirillales* frame to a *Rhodocyclus* ancestor.

##### *Gemmatimonas (Gemmatimonadales)* 

Most significantly, *Gemmatimonas phototrophica* was found at the deepest branching point in the RNA tree, which placed this bacterium apart from all other phototrophic purple bacteria and also the phototrophic green bacteria. However, with the *Proteobacteria,* it shared the type-2 photosystem. In the PS tree, it formed a distinct line that split off at the deepest branching point from the *Burkholderiales* and was distantly linked to *Rubrivivax* (*Betaproteobacteria*). This was an indication that the photosynthetic roots of *Gemmatimonas* were associated with the ancient roots of the phototrophic *Burkholderiales*. If we exclude the acquisition of a foreign 16S rRNA, the most likely explanation for this discrepancy would be the acquirement of the photosynthesis genes by an early ancestor of *Gemmatimonas*, as suggested by Zeng et al. [26]. This event should have preceded the branching divergence of the *Burkholderiales.*

##### *Alphaproteobacteria* 

The phototrophic *Alphaproteobacteria* formed the most fragmented and diverse array of groups in the PS tree with representatives of the six orders *Rhodospirillales, Rhizobiales, Sphingomonadales, Rhodobacterales, Caulobacterales,* and *Rhodothalassiales*. Most significantly, the *Rhodobacterales,* together with *Sphingomonadales* and *Brevundimonas* (*Caulobacterales*), formed a major branch, according to BchXYZ-PufHLM, which was clearly distinct from all other phototrophic *Alphaproteobacteria* (Figure 1, Figure 3 and Figure 4). A deep branching point separated the *Sphingomonadales* and *Brevundimonas* from the *Rhodobacterales*. The relations of other *Alphaproteobacteria*, however, were more problematic because most of the species had long-distance lines with deep branching points and only a few species arranged in stable groups that could be recognized in both PS tree and RNA tree.

*Rhodobacterales.* The most recent and shallow divergences were seen in the phylogeny of the *Rhodobacterales*, which, in contrast to most other phototrophic *Alphaproteobacteria,* appeared as a young group that had differentiated later than others and was well established as a group in PS tree and RNA tree. It diversified quite fast in evolutionary terms and now represents one of the largest orders of phototrophic bacteria known. The following groups of *Rhodobacterales* were formed in the PS tree. With the exception of the *Rhodobacter* group and the *Rhodovulum* group they represent aerobic phototrophic bacteria.

- *Rhodovulum* group: According to BchXYZ and BchXYZ-PufHLM, the *Rhodovulum* group was well recognized. *Rhodobaculum claviforme* appeared distantly associated with this group and, like the *Rhodovulum* species, had PufC (Supplementary Table S1). However, in the RNA tree, *Rhodobaculum claviforme* clustered with *Rhodobacter* species.
- *Rhodobacter/Rhodobaca* group: This group contained *Rhodobacter* and *Rhodobaca* species together with *Cereibacter changlensis* and was supported by all considered trees (BchXYZ-PufHLM, BchXYZ, RNA tree). The reaction center cytochrome PufC was absent (Supplementary Table S1). Quite remarkable *Rhodobaculum claviforme* was included in this group according to the RNA tree only.
- *Loktanella/Sulfitobacter* group: This group included species of *Loktanella*, *Sulfitobacter*, *Planktomarina,* and *Roseisalinus* and distantly linked also *Nereida ignava*. It was supported by BchXYZ-PufHLM and lacked PufC (Supplementary Table S1). According to the RNA tree, this group was not well supported, and *Roseobacter* but not *Roseisalinus* was included.
- *Roseobacter/Roseivivax* group: This group contained species of *Roseobacter*, *Roseivivax*, *Salipiger*, and *Roseovarius*. In line with the PS tree, PufC was present in all species, including *Roseobacter*. The RNA tree excluded *Roseobacter* from this group.
- *Dinoroseobacter/Jannaschia* group: *Dinoroseobacter shibae*, *Jannaschia aquamarina, Thalassobacter stenotrophicus,* and *Roseibacterium elongatum* formed a group of poorly linked bacteria, which did not fit into any of the aforementioned groups. All four species had PufC. Within the RNA tree, there was only weak support for this group (Figure 1).

*Sphingomonadales.* The *Sphingomonadales* formed a consistent lineage of aerobic phototrophic bacteria within all considered phylogenetic trees. *Sphingomonadaceae* with *Sphingomonas* and *Novosphingobium* species (likely also *Blastomonas,* see [3]) were forming one sub-branch and the *Erythrobacteraceae* with *Erythrobacter* and *Porphyrobacter* species (likely also *Erythromicrobium,* see [3]) a second one. There was support for the inclusion of *Erythrobacter marinus* into the *Sphingomonas* group from BchXYZ and BchXYZ-PufHLM phylogeny. In addition, *Erythrobacter marinus* contained PufC like *Sphingomonas* and *Novosphingobium* species but unlike other *Erythrobacteraceae*. According to the RNA tree, *Erythrobacter marinus* clustered with other *Erythrobacter* species, however, with low confidence in its position.

*Brevundimonas (Caulobacterales). Brevundimonas subvibrioides* represented an aerobic phototrophic bacterium, which clearly but distantly was linked to the *Sphingomonadales* branch according to the PS tree and RNA tree. *Brevundimonas* lacked PufC as the *Erythrobacteraceae* did. The deep branching point of *Brevundimonas* in the PS tree indicated that it was closest to the common ancestor of this branch.

The *Rhodobium/Hoeflea* group. A most deeply branching stable lineage in the PS tree was found within the radiation of the *Alphaproteobacteria* and was represented by the *Rhodobium/Hoeflea* group with *Rhodobium orientis*, *Hoeflea phototrophica*, *Labrenzia alexandrii,* and *Oceanibaculum indicum* (Figure 2 and Figure 3). Despite the formation of a coherent group according to the PS tree, the species had different, though unsupported positions in the RNA tree (Figure 1 and Figure 4). According to 16S rRNA, *Hoeflea phototrophica* (*Rhizobiales*, *Phylobacteriaceae*) had a deeply branching unsupported position; *Labrenzia alexandrii* (*Rhodobacterales*, *Rhodobacteraceae*) also had an unsupported position that was linked at the basis to *Brevundimonas* and the *Spingomonadales*; *Rhodobium orientis (Rhizobiales, Rhodobiaceae)* was found together with *Afifella* in a poorly rooted distinct branch; *Oceanibaculum indicum* (*Rhodospirillales, Rhodospirillaceae*) appeared distantly associated with *Rhodocista*, *Skermanella,* and the *Acetobacteraceae* (Figure 1). The photosynthesis of the *Rhodobium/Hoeflea* group represented one of the most ancient lines among the purple bacteria, and the most recent divergence (between *Labrenzia* and *Oceanibaculum*) was rooted much deeper as the basic divergence of the *Rhodobacterales* branch (Figure 3). In addition, there is no close relative to the photosynthesis system among other known phototrophic bacteria, which is a clear indication of the very ancient origin of photosynthesis in this lineage of phototrophic bacteria. If we do trust the phylogenetic reliability of the 16S rRNA system, we should assume quite early genetic transfers of major parts or the complete photosystem from an ancient ancestor within the *Rhodobium* lineage to the other bacteria. Alternatively, as the species of this branch formed poorly rooted lines in the RNA tree, the differences between PS and RNA tree might be explained by unresolved relationships and not correctly rooted positions of these bacteria in the RNA tree.

The *Rhodopseudomonas*/*Bradyrhizobium* group. In the PS tree, the *Rhodopseudomonas/Bradyrhizobium* group formed one of the most deeply branching lines distinct from other *Rhizobiales*. Both *Rhodopseudomonas* and *Bradyrhizobium* lacked PufC. According to 16S rRNA phylogeny, *Rhodopseudomonas* and *Bradyrhizobium* formed a sister branch to the photosynthetic *Methylobacterium* species, distant to other *Rhizobiales* (*Blastochloris* and *Rhodoplanes*, *Methylocella* and *Rhodoblastus, Prosthecomicrobium* and *Rhodomicrobium*).

The *Rhodopila* group. Another distinct group was represented by the *Acetobacteraceae* and supported by both RNA tree and PS tree with species of *Rhodopila*, *Acidiphilum*, *Paracraurococcus,* and *Rubritepida*.

The *Rhodospirillum* group. According to the PS tree, species of *Rhodospirillum*, *Pararhodospirillum,* and *Roseospirillum parvum* formed a group to which *Rhodospira trueperi* appeared distantly linked. In the RNA tree, *Caenispirillum* was included in this group, while in the PS tree, it had a separate position and formed a branch together with *Rhodovibrio* species, which, in turn, appeared as an isolated line at the basis of the *Alphaproteobacteria* within the RNA tree.

In addition to these groups, several separate lineages were represented by single genera of *Fulvimarina, Rhodothalassium*, *Prosthecomicrobium,* and *Afifella* in both PS and RNA trees (Figure 1 and Figure 3). Thus, their phylogenetic positions remained unclear. While *Methylocella* specifically associated with *Rhodoblastus* in both RNA tree and PS trees, the following groupings were not well supported or had different positions in PS tree and RNA tree:

- *Rhodocista centenaria* and *Skermanella* species showed up jointly in the RNA tree with the Acetobacteraceae as a sister branch, while both formed a deeply rooted unsupported branch in the PS tree.
- *Rhodomicrobium* formed a distinct lineage within the *Rhizobiales* in the RNA tree but, according to the PS tree, separated from other *Rhizobiales* together with *Rhodoplanes* in a distinct deeply branching but unsupported line.
- *Blastochloris* separated from other *Rhizobiales* in the PS tree and formed an unsupported isolated line together with the bacteriochlorophyll-b containing *Rhodospira trueperi*, while it was included in a major branch of *Rhizobiales* in the RNA tree.

### 3.4. Distribution of PufC

The cytochrome associated with the photosynthetic reaction center is an important component in many of the PS-II type photosynthetic bacteria. As a more peripheral part of the photosynthetic reaction center, the cytochrome may be more easily replaced by alternative electron transport systems, and this obviously happened in a number of phylogenetic lineages of the *Alphaproteobacteria* and *Betaproteobacteria* (Supplementary Table S1). The general presence of the reaction center cytochrome PufC in phototrophic purple bacteria and the absence in quite a few distinct groups of the *Alphaproteobacteria* and *Betaproteobacteria* strongly suggested that PufC independently has been lost several times. PufC was absent in *Rhodoferax fermentans* (but present in the related *Rhodoferax antarcticus*), in *Rhodospirillum rubrum* (but present in the related *Pararhodospirillum photometricum*), in *Bradyrhizobium* and *Rhodopseudomonas* species (but present in other *Rhizobiales*), in *Brevundimonas*, *Porphyrobacter,* and *Erythrobacter* species (but present in *Erythrobacter marinus* and *Sphingomonadaceae*). It was also absent in one of the major *Rhodobacterales* branches of the PS tree, including the *Rhodobacter/Rhodobaca* group and the *Loktanella/Sulfitobacter* group. The presence/absence of PufC in species of the *Alphaproteobacteria* was congruent with the photosynthesis phylogeny. The presence of PufC supported the inclusion of *Rhodobaculum* into the *Rhodovulum* group, and its absence in *Roseisalinus* was in accord with its inclusion into the *Loktanella/Sulfitobacter* group according to the PS tree. Following the PS tree and the presence of PufC, *Erythrobacter marinus* fitted into the *Sphingomonadaceae* (rather than into the *Erythrobacteraceae*).

### 3.5. Phylogenetic Aspects of Aerobic Anoxygenic Photosynthesis

As oxygen was absent from the earth during the first billion years of life, in which the basic concepts of photosynthesis are expected to have evolved and as oxygenic photosynthesis using two different consecutive photoreactions is considered a late event in the evolution of photosynthesis, the roots of anoxygenic, as well as oxygenic photosynthesis, are to be found in the ancient anoxic environments [30,31]. We assume that with the onset of oxygenic photosynthesis, the basic concepts of photosynthesis, as known today, have already been established. In fact, oxygen evolution by photosynthesis using water as electron source is dependent on the use of two different consecutive photosynthetic reactions that have evolutionary ancestors among anoxygenic phototrophic bacteria: a type-I photosynthesis in ancestors of *Heliobacteria*, *Chlorobi*, and *Chloracidobacterium* and a type-II photosynthesis in ancestors of *Chloroflexi* and all phototrophic purple bacteria [31].

The appearance of oxygenic photosynthesis approx. 3 billion years ago was a revolution in ecology. It drastically changed the environmental conditions on earth, and over approx. 2 billion of years caused the gradual increase of the atmospheric oxygen content to the actual level [32]. Quite likely, during this transition period, the radiation of the purple bacteria diverged to its full extension.

During adaptation to oxic conditions, quite a number of anoxygenic phototrophic purple bacteria may have gained the ability to perform under both anoxic and oxic conditions by maintaining the strict regulation of biosynthesis of the photosynthetic apparatus and its repression by oxygen. An example of such bacteria is found in *Rhodobacter* species performing anoxygenic photosynthesis under anaerobic conditions in the light and aerobic respiration under oxic conditions in the dark [33,34,35,36]. During further evolution, some of these phototrophic bacteria may have lost the ability to build up the photosynthetic apparatus in the absence of oxygen and, in contrast, required oxygen for the formation of the photosynthetic apparatus [37]. In bacteria, such as the anaerobic phototrophic *Rhodobacterales*, that have already been adapted to arrange themselves with certain levels of oxygen, this could have been a small step in modifying the oxygen-response in the formation of the photosynthetic apparatus. As a result, in various phylogenetic branches, aerobic anoxygenic phototrophic bacteria have evolved, of which *Erythrobacter* and *Roseobacter* species at present are the most well-known examples [37,38,39].

Aerobic representatives of phototrophic purple bacteria that require oxygen for the synthesis of bacteriochlorophyll and the photosynthetic apparatus were found in a number of well-defined groups. The *Haliaceae* of the *Gammaproteobacteria* and the *Erythrobacteraceae* and *Sphingomonadaceae* of the *Sphingomonadales* at present exclusively contain aerobic representatives. In addition, isolated lines of single representatives of aerobic phototrophic bacteria were found with species of *Fulvimarina*, *Gemmatimonas, Polynucleobacter,* and *Brevundimonas*. In the *Rhodobium/Hoeflea* group, several aerobic phototrophic bacteria joined the anaerobic phototrophic *Rhodobium*. In the *Acetobacteraceae,* several aerobic representatives were found together with the anaerobic phototrophic *Rhodopila*. A larger group of the aerobic phototrophic *Rhizobiales* with *Methylobacterium*, *Methylocella*, *Prosthecomicrobium,* and *Bradyrhizobium* was related to the anaerobic phototrophic *Rhodopseudomonas* and *Rhodoblastus* species. Among the *Rhodobacterales,* the groups around *Loktanella/Sulfitobacter*, *Dinoroseobacter/Jannaschia,* and *Roseivivax/Roseovarius* represented branches with aerobic species.

With the exception of the aerobic phototrophic *Rhodobacterales*, most of the aerobic phototrophic bacteria represented ancient phylogenetic lineages. This was especially the case for the *Sphingomonadales*, *Brevundimonas* (*Caulobacterales*), the *Haliaceae,* and those within the *Rhodobium/Hoeflea* group. As traces or small levels of oxygen already were present at the time when anaerobic phototrophic purple bacteria presumably diversified approx. 2.5 billion years ago, the aerobic phototrophic purple bacteria could have developed in parallel, which would explain the deep divergence of some of the lineages of aerobic phototrophic *Proteobacteria*.

The pattern of distribution of aerobic phototrophic bacteria among the phototrophic purple bacteria strongly suggested that aerobic phototrophic purple bacteria evolved from anaerobic ancestors in independent and multiple events. The deep branching points of some lineages indicated their early divergence from the anaerobic phototrophic ancestors. The phylogeny suggested that in the *Rhodobium/Hoeflea* group, photosynthesis of aerobic representatives evolved from an anaerobic ancestor with a common root with *Rhodobium*. Known representatives of aerobic phototrophic *Sphingomonadales*, *Cellvibrionales (Haliaceae*), *Caulobacterales* (*Brevundimonas*), and *Gemmatimonas* could be present-day survivors of ancient anaerobic phototrophic relatives not known so far or extinct.

The development of aerobic phototrophic *Rhodobacterales* was considered to be a more recent event, as these bacteria and their photosynthesis were much younger compared to most other phototrophic bacteria (Figure 3). It has been calculated by molecular clock calculations using sequences of representative genes that the divergence of the last common ancestor of *Roseobacter* and *Rhodobacter* dates to approx. 900 Myr ago (+/- 200 Myr) [40]. At that time, the oxygen content of the earth’s atmosphere almost had reached present-day levels [32], and it is tempting to assume that aerobic phototrophic lineages branched off from anaerobic phototrophic *Rhodobacterales* under these conditions. Though this is considered the more likely scenario, alternatively, the common ancestor of the *Rhodobacterales* could have been an aerobic phototrophic bacterium. This scenario could find support in the common roots of *Rhodobacterales* with the aerobic phototrophic *Sphingomonadales* and *Brevundimonas* but implies that the ancestors of *Sphingomonadales* and *Brevundimonas* also were aerobic phototrophic bacteria, which is not necessarily the case. It would also imply that aerobic phototrophic *Rhodobacterales* transformed back to perform anaerobic photosynthesis, which from an evolutionary and ecological perspective appears quite unlikely. Therefore, we assume that aerobic phototrophic representatives of *Rhodobacterales*, *Sphingomonadsales,* and *Brevundimonas* evolved independently from anaerobic phototrophic ancestors.

### 3.6. General Aspects

Traditionally and especially due to the pioneering work of Carl Woese [25], the 16S rRNA gene sequence has been established as the basic tool for the analysis of bacterial phylogenies. Though the sequence information contained in this molecule is of limited size (approx. 1400 nt), it is considered as particularly conservative in evolutionary terms. In consequence, 16S rRNA gene sequences still are used as a backbone for phylogenetic considerations, although limitations are to be expected due to the comparable small sequence information and restricted resolution. Further limitations may be due to multiple changes in individual sequence positions and insertions/deletions over time, which could blur the phylogenetic roots in particular of the deep branching lineages.

In this context, it was quite remarkable that some of those species/groups that revealed the most obvious differences between the PS tree and RNA tree also showed the deepest branching points within the PS tree. In fact, a number of those species and branches that were not congruent with respect to RNA and PS phylogeny had statistically poorly supported positions in either one or both of the trees. This was especially relevant for most of the *Alphaproteobacteria*, which appeared—with the remarkable exception of the *Rhodobacterales—*to be the most ancient group of phototrophic *Proteobacteria*.

Despite the uncertainty in the resolution of the very deeply branching lineages, the transfer of photosynthesis genes could explain several of the discrepancies between the PS and RNA trees. Such mechanisms have been postulated earlier [26,29], and genetic exchange could have occurred repeatedly in the early ages of photosynthesis in *Proteobacteria*. Examples of such possible exchange events during early diversification of the phototrophic purple bacteria may be found in *Gemmatimonas phototrophica*, the *Rhodobium/Hoeflea* group, and the *Betaproteobacteria* with different events of *Rhodocyclus* and *Rubrivivax* and their relatives.

The situation was different within the *Rhodobacterales*, which is a well-resolved group with a clear distinction from other *Proteobacteria*. It is the youngest diversification within the phototrophic *Proteobacteria*. For this group, lateral gene transfer has been demonstrated [41]. It could, in fact, be shown that the photosynthetic gene cluster in several genomes, including *Sulfitobacter* and *Roseobacter* species, is located on a plasmid, which enforces the genetic exchange of the whole cluster [41]. Several of such exchanges could explain the divergences between photosynthesis phylogeny and RNA phylogeny among the *Rhodobacterales* [41]. Despite the established gene transfer inside the *Rhodobacterales*, it appears highly unlikely that the whole group received the photosynthetic gene cluster by lateral transfer from an external donor. The long phylogenetic distance to most other photosynthesis systems and the basically good correlation of RNA and PS phylogeny in regard to relations of *Sphingomonadales*, *Brevundimonas,* and *Rhodobacterales* precludes the transfer from any other known phototrophic lineage. As we have no knowledge of the existence of similar gene transfer agents in other phototrophic bacteria, this kind of genetic exchange of the complete photosynthetic gene cluster could be a late acquisition and unique to the *Rhodobacterales*. However, it would be interesting to study the situation in *Erythrobacter marinus,* which could have similarly received its photosynthesis genes from a relative of the *Sphingomonadaceae* branch.

## 4. Conclusions

The immense phylogenetic diversity of photosynthetic prokaryotes was demonstrated by the wide systematic range of these bacteria. Bacteria considered in this communication were cultured representatives from six phyla (the cyanobacteria were not considered here) with 15 orders, 27 families, and 90 genera. The most ancient representatives of the phototrophic bacteria, the first that made bacteriochlorophyll (chlorophyllide reductase, BchXYZ) and performed photosynthesis were the phototrophic green bacteria, in particular, those with a type-I photosystem (*Chlorobi*, *Heliobacterium*, *Chloracidobacterium*) (Figure 1 and Figure 2). Among those with a type-II photosystem, the *Chloroflexi* have by far the most ancient roots [3], and *Proteobacteria*, together with their photosystem, diversified to the present-day forms much later (Figure 1 and Figure 3). There was an apparent large gap in the evolution of photosynthesis in the phototrophic green bacteria and in the *Proteobacteria*. This is an indication for the loss of early stages of the photosystem present in the Proteobacteria (Figure 1 and Figure 2).

Today phototrophic *Proteobacteria* are by far the most diverse and the most abundant in the environment and have to be considered the most successful to adapt to the largely oxic environment. If we consider that the basic divergences within the *Rhodobacterales* (e.g., the separation of *Rhodobacter* and *Roseobacter*) have occurred approx. 1 billion years ago [40] and that the first photosynthetic prokaryotes have evolved approx. 3.2–3.5 billion years ago, it is reasonable to conclude from the phylogeny of photosynthesis that phototrophic *Proteobacteria* appeared around 2–2.5 billion years ago. If we use these rough estimates as a guide for the interpretation of the phylogenetic relations of photosynthesis, we can conclude that the ancestors of the green bacteria dominated the field over approx. a billion years and quite likely ancestors of the strictly anaerobic *Chlorobi* played a prominent role in the sulfur oxidation during this time. The *Chlorobi* maintained their strict phototrophic and also an anaerobic way of life up to today and consequently are pushed back to the few anoxic/sulfidic ecological niches that receive light. In the early ages also, the photosystem type-II originated and presumably soon separated into a system represented by our present-day *Chloroflexi* and a system that developed later within the phototrophic *Proteobacteria*. If we assume a common origin of the photosystem type-II in *Chloroflexi* and *Proteobacteria*, the system, as we know it from the *Proteobacteria*, is an advanced stage of a parallel development that diversified together with these bacteria much later. Ancient forms that could represent a link between the two type-II photosystems, apparently, were extinct or survivors have not yet been detected.

The most ancient roots of photosynthesis among *Proteobacteria* are found in the *Alphaproteobacteria* (excluding *Rhodobacterales*) and *Betaproteobacteria* with often unsupported deep divergences and long lines to the present-day representatives, the species/strains studied. Photosynthesis in *Gammaproteobacteria* diversified significantly later with the origin of the *Ectothiorhodospira* group, predating that of the others (*Halorhodospira*, *Chromatiaceae*, *Cellvibrionales*). As the photosynthesis phylogeny in general terms was congruent with the RNA phylogeny (Figure 1, Figure 2, Figure 3 and Figure 4), it was concluded that this type-II photosystem diversified together with the *Proteobacteria*.

Compared to the phylogenetic depth and the systematic width found in the radiation of the *Alphaproteobacteria*, relatively few genera are known of these bacteria, with the exception of *Rhodobacterales*. The *Rhodobacterales,* on the other hand, represent the most recently diverged group. These bacteria apparently are the most successful to live in our mostly oxic world today, are most versatile in their metabolism, are well adapted to live in the oxic environment, and represent one of the largest orders of phototrophic bacteria living today. A second large gap in the evolution of photosynthesis is, in fact, seen between the *Rhodobacterales* and all other *Proteobacteria* (Figure 3). Another large group, which is clearly separated from the others, but diversified earlier than the *Rhodobacterales* is represented by the *Chromatiales*. These bacteria characteristically are adapted to the borderline between the anoxic/sulfidic and the oxic environment, have found ecological niches over billions of years and survived successfully until today.

The phototrophic bacteria included in this investigation were representatives of most of those that are known and in laboratory culture today. Therefore, the presented data gave a comprehensive basis of the phylogeny of anoxygenic photosynthesis, although the view was limited due to the fact that all those that have escaped cultivation attempts or for other reasons have not been cultured could not be considered. As genetic studies with communities of phototrophic purple bacteria from marine coastal sediments based on the PufLM metagenomic diversity demonstrated that many of those present (or close relatives thereof) already had been cultivated [7], it was concluded that a great part of those out in nature have already been identified, at least from coastal marine sediments. Nevertheless, we will certainly continue finding new species and phylogenetic lines of phototrophic bacteria, in particular when unstudied or poorly studied locations and environments are investigated. Comprehensive metagenome studies on a great number of environmental communities might even detect missing links of photosynthesis evolution.

## Supplementary Materials

The following are available online at https://www.mdpi.com/2076-2607/7/11/576/s1.
